# Gender Variations in the Oral Microbiomes of Elderly Patients with Initial Periodontitis

**DOI:** 10.1155/2021/7403042

**Published:** 2021-11-23

**Authors:** Jie Zhao, Ying-Hui Zhou, Ya-Qiong Zhao, Yao Feng, Fei Yan, Zheng-Rong Gao, Qin Ye, Yun Chen, Qiong Liu, Li Tan, Shao-Hui Zhang, Jing Hu, Marie Aimee Dusenge, Yun-Zhi Feng, Yue Guo

**Affiliations:** ^1^Department of Stomatology, The Second Xiangya Hospital of Central South University, Changsha, 410011 Hunan, China; ^2^National Clinical Research Center for Metabolic Diseases, Hunan Provincial Key Laboratory of Metabolic Bone Diseases, Department of Metabolism and Endocrinology, The Second Xiangya Hospital of Central South University, Changsha, 410011 Hunan, China; ^3^Hunan Key Laboratory of Oral Health Research & Hunan 3D Printing Engineering Research Center of Oral Care & Hunan Clinical Research Center of Oral Major Diseases and Oral Health & Xiangya Stomatological Hospital & Xiangya School of Stomatology, Central South University, Changsha 410008, China

## Abstract

Periodontitis is a globally prevalent disease that imposes a functional and aesthetic burden on patients. The oral microbiome influences human health. The aim of this study was at assessing gender variation in the subgingival bacterial microbiome of elderly patients with initial periodontitis and to determine the causes of this variation. Twelve males and twenty females (range 50–68 years old) with initial periodontitis provided subgingival plaque samples. 16S rRNA gene sequencing, QIIME-based data processing, and statistical analyses were carried out using several different analytical approaches to detect differences in the oral microbiome between the two groups. Males had higher Chao1 index, observed species, and phylogenetic diversity whole tree values than females. Analysis of *β*-diversity indicated that the samples were reasonably divided by the gender. The linear discriminant analysis effect size showed that the most representative biomarkers were the genus *Haemophilus* in males, whereas the dominant bacteria in females were *Campylobacter*. Kyoto Encyclopedia of Genes and Genomes analysis showed that predicting changes in the female oral microbiota may be related to the immune system and immune system diseases are the main factor in males. These data suggest that gender may be a differentiating factor in the microbial composition of subgingival plaques in elderly patients with initial periodontitis. These results could deepen our understanding of the role of gender in the oral microbiota present during initial periodontitis.

## 1. Introduction

The human oral cavity is composed of unique niches colonized by a variety of microorganisms, including bacteria, fungi, viruses, and archaea, which create a rich microbial community known as the oral microbiome [1]. These communities of microbes are highly dynamic and responsible for various functions that can both maintain and deplete oral health. Oral microbiomes often exist in a homeostatic equilibrium with the host, but under certain conditions, this equilibrium is disturbed. Oral microbiome dysbiosis can cause a wide range of systemic illnesses, including diabetes [2], cancer [3], Alzheimer disease [4], rheumatoid arthritis [5], and cardiovascular disease [6]. For example, a higher pancreatic cancer risk has been associated with the enrichment of *Porphyromonas gingivalis* and *Aggregatibacter actinomycetemcomitans* [7]. Furthermore, oral *P. gingivalis* infection in mice results in brain colonization of an Alzheimer's disease model and increased production of A*β*1-42, a component of amyloid plaques [8]. Oral microbiome dysbiosis also directly affects oral health, potentially causing periodontitis and dental caries, two of the most prevalent and typical microbially induced disorders worldwide. Such periodontal disease will also affect systemic health, including the immune system [9] and digestive system [10].

Age correlates with the severity of periodontal disease and dental caries. Because of the decline in immune system function and low salivary flow rates brought on by the natural advancement of age, there is a clinical increase in the prevalence and severity of periodontitis and dental caries in older adults. Age is also an important factor in oral microbiome dysbiosis. Rodenburg et al. found that the prevalence of subjects colonized by *A. actinomycetemcomitans* appeared to be age related, as it decreased with increasing age [11]. In addition, the incidence and severity seem to have a gender predilection with the incidence and severity of periodontal disease appearing to be greater in males than in females [12, 13]. Lira-Junior et al. found that male gender presented higher counts of 16 bacteria in saliva than female gender, which may be related to the slightly worse periodontal condition in male than in female [13]. Furthermore, the NHANES III survey has demonstrated, in most age groups, a higher prevalence and greater extent of attachment loss, as well as a higher prevalence of periodontitis and deeper probing depths in males compared to females [14]. However, females have been shown to be more susceptible to caries [15]. Thus, gender can logically be assumed to be an important factor affecting the oral microbiome.

The immunological response produced by the oral microbiome is important and complex. Tissue homeostasis is maintained by innate immunity, which prevents destruction of the periodontal tissue. Severe periodontitis is characterized by neutropenia, agranulocytosis, neutrophil adhesion, deficiencies in chemotaxis, and diseases affecting lysosomal degranulation [16]. The cellular infiltrate in human gingivitis is primarily composed of T helper (Th) cells. The phenotypes of Th cells are directed by phagocytic antigen-presenting cells, including dendritic cells (DCs) and Langerhans cells [17]. The immune response in the gingiva, during an undefined transition, switches from neutrophil recruitment and activation to pathogenic bacteria to chronic infiltration of T and B cells and plasma cells [18] that induces vascular prolifera and the destruction of connective tissue. At the same time, sex steroids are related to the immune system. Youssef and Stashenko indicated that estrogen directly stimulates IL-1 production by macrophages, leading to heightened immune responses and the resistance of females to disseminating dentoalveolar infections [19]. In contrast, androgen has been reported to play a suppressive role in the immune response and to act as a potential promoter of tumor growth and protector from autoimmune diseases [20]. Androgen-deficient male C57BL/6 mice were shown to be significantly more susceptible to endotoxic shock, possibly due to the significantly higher expression of surface TLR4 on macrophages [21].

Therefore, the aim of the present study was to assess gender variation in the subgingival bacteria microbiome of elderly patients with initial periodontitis and determine the causes of this variation. Specifically, we examined whether bacterial taxa were differentially abundant, whether gender variation exists in the *α*- and *β*-diversity using high-throughput sequencing data, and whether the variation is related to the immune system.

## 2. Materials and Methods

### 2.1. Study Population and Sample Data

All participants were recruited from The Second Xiangya Hospital of Central South University in Changsha, China. The Ethics Committee of The Second Xiangya Hospital of Central South University provided ethics and regulatory approval. Verbal and written informed consent were obtained from all participants. The clinical trial registration number is ChiCTR2100046828.

The participants included in this study were 50 to 68 years old [22–24], with ≥15 teeth present and at least one natural tooth in all six sections. Exclusion criteria included smoking, infection, trauma or recent surgery, pregnancy, systemic disease (e.g., heart failure, autoimmune disease, musculoskeletal disorders, and malignancy), the use of antibiotics or immunomodulators in the previous 30 days, periodontal treatment in the previous 6 months, or oral disease (e.g., oral lichen planus, ulcer, oral leukoplakia, and pharyngitis). Questionnaires were used to obtain data from all patients who agreed to serve as subgingival plaque donors. We enrolled 32 patients with initial (stage I) periodontitis, including 12 males and 20 females. Before collecting samples, sites were air dried. The stage of periodontal disease was determined according to the 2017 World Workshop on the Classification of Periodontal and Peri-Implant Diseases and Conditions “Staging and grading of periodontitis: Framework and proposal of a new classification and case definition” [25]. The clinical attachment level (CAL) of the most severe site was recorded for each tooth; 1–2 mm was defined as stage I, 3–4 mm as stage II, and ≥5 mm as stage III. The number of missing teeth was also staged, with no missing teeth indicating stages I and II, ≤4 teeth missing stage III, and ≥5 teeth missing stage IV. Notably, the reasons for the missing teeth were not considered. The complexity of management was evaluated by reclassifying stage II patients as stage III if the maximum probing pocket depth (PPD) was ≥6 mm, and stage III patients as stage IV if <20 teeth or 10 opposing pairs remained. The subgingival plaques of the four first molars were scraped at the bottom of the periodontal pocket using a sterile Gracey scraper. The samples were transferred to PBS, immediately frozen, and maintained at −80°C.

### 2.2. DNA Extraction and Sequencing of the 16S rRNA V3-V4 Region

Based on the manufacturer's instructions, total genomic DNA was extracted using a DNA Extraction Kit (DNeasy PowerSoil Kit, QIAGEN). The extracted DNA was quantified using a NanoDrop 2000 spectrophotometer (Thermo Fisher Scientific, Waltham, MA, USA) and the quality confirmed by agarose gel electrophoresis. The universal PCR primers 343 F (5′-TACGGRAGGCAGCAG-3′) and 798 R (5′-AGGGTATCTAATCCT-3′) were designed to amplify the V3-V4 variable regions of bacterial 16S rRNA genes. 16S rRNA gene sequencing was completed by OEBiotech (Shanghai, China). PCR was performed using the following program: initial denaturation at 94°C for 5 min, 26 cycles of denaturation at 94°C for 30 s, annealing at 56°C for 30 s, elongation at 72°C for 20 s, and a final extension at 72°C for 5 min. Amplicons were purified using AMPure XP beads (Agencourt, Beckman Coulter, USA) and quantified using the Qubit dsDNA Assay Kit (Life Technologies, USA) following the manufacturers' instructions. Purified amplicons were pooled for sequencing. The raw reads were generated by Illumina MiSeq (V1.9.1), and the sequences were processed and analyzed in QIIME (v1.9.1). Briefly, the effective sequences were clustered to the same OTUs with ≥97% identity using VSEARCH (v.2.4.2) [26]. Next, the annotation information of representative sequences of each OTU was analyzed using the RDP classifier Naive Bayesian method [27] and the SILVA database.

### 2.3. Bioinformatics and Statistical Analysis

The *α*-diversity indices (Chao, Shannon, Simpson, Good's coverage, and phylogenetic diversity (PD) index) were calculated at 97% identity by the Wilcoxon rank-sum test. Samples from different groups were compared by Student's *t*-test and the Kruskal-Wallis test. Beta diversity analysis was performed by principal coordinates analysis (PCoA) based on unweighted UniFrac distances at the OTU level. Principal component analysis (PCA) was also conducted. The analysis of nonmetric multidimensional scaling (NMDS) based on weighted UniFrac distances was conducted to compare different groups. The relative abundance of predominant bacteria was compared between different groups using the Wilcoxon rank-sum test. The core microbiome was defined at the species level using a Venn diagram. We performed linear discriminant analysis (LDA) of effect size (LEfSe) to define biomarkers in the four groups. The logarithmic LDA score threshold for distinguishing features was 2.0. The functional content from the16S rRNA gene sequences was predicted using phylogenetic investigation of communities by reconstruction of unobserved states (PICRUSt) software [28] by linking taxonomic information to the Kyoto Encyclopedia of Genes and Genomes (KEGG) annotations of the reference genome. Differences were considered significant when *P* < 0.05. SPSS 25.0 software was used for statistical analyses.

## 3. Results

### 3.1. Characteristics of Selected Patients and OTU Basic Analysis

**Figure 1:**
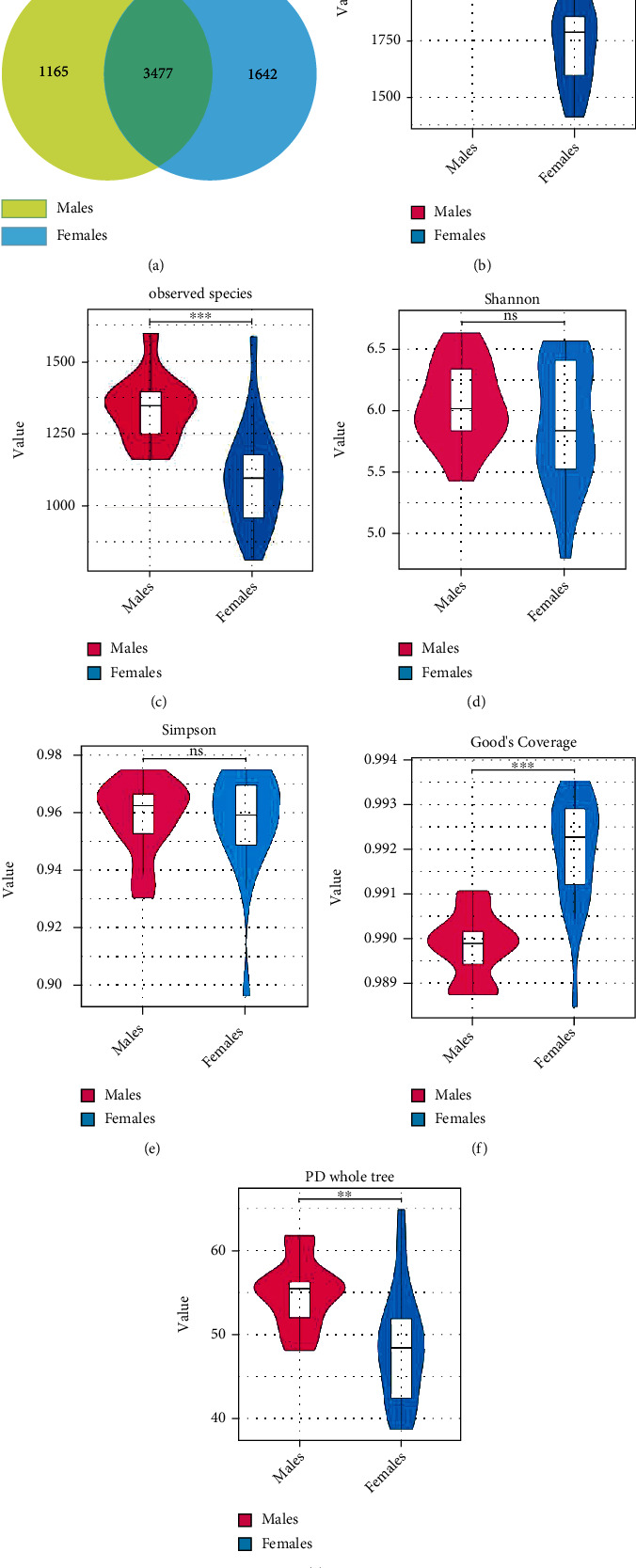
Venn diagram and *α*-diversity analysis of elderly males and females with stage 1 periodontitis. (a) Venn diagram based on operational taxonomic units (OTUs). (b–g) Violin plots comparing *α*-diversity indices (Chao1, observed species, Shannon index, Simpson index, Good's coverage, and PD whole tree) between males and females. ^∗∗∗^*P* < 0.001; ^∗∗^*P* < 0.01; ns: not significant.

Patient age (*n* = 32) ranged from 50 to 68 years. The male patients had a mean age of 54 years (range 51–60 years) and female patients 58.6 years (range 50–68 years). There was no statistically significant difference in age between male and female (*P* > 0.05, Table 1). The Venn diagram of differences in the OTUs (Figure 1(a)) showed that males and females had 3477 common OTUs. However, 1642 OTUs were higher in females and 1165 in males.

### 3.2. Diversity Analyses

Alpha diversity reflects the abundance and diversity of microbial communities. The Chao1 index and observed species were used to calculate community richness, whereas the Shannon and Simpson indices were able to evaluate community diversity. Compared to females, males had a significantly higher number and/or diversity of taxa based on the Chao1 index (*P* < 0.01, Figure 1(b)) and observed species (*P* < 0.01, Figure 1(c)). No significant difference was apparent between the two groups in the Shannon (6.37 versus 6.56, Figure 1(d)) and Simpson (0.96 versus 0.97, Figure 1(e)) indices. Higher Good's coverage index values were associated with a higher probability of species being measured in the sample. Although Good's coverage was significantly different between the two groups (*P* < 0.01), both groups reached 0.99 for the depth of sequence representing the majority of bacterial species in the plaque samples. The PD whole tree index reflects the relationships of species within the community and was found to decrease in females compared to males (Figure 1(g)).

**Figure 2:**
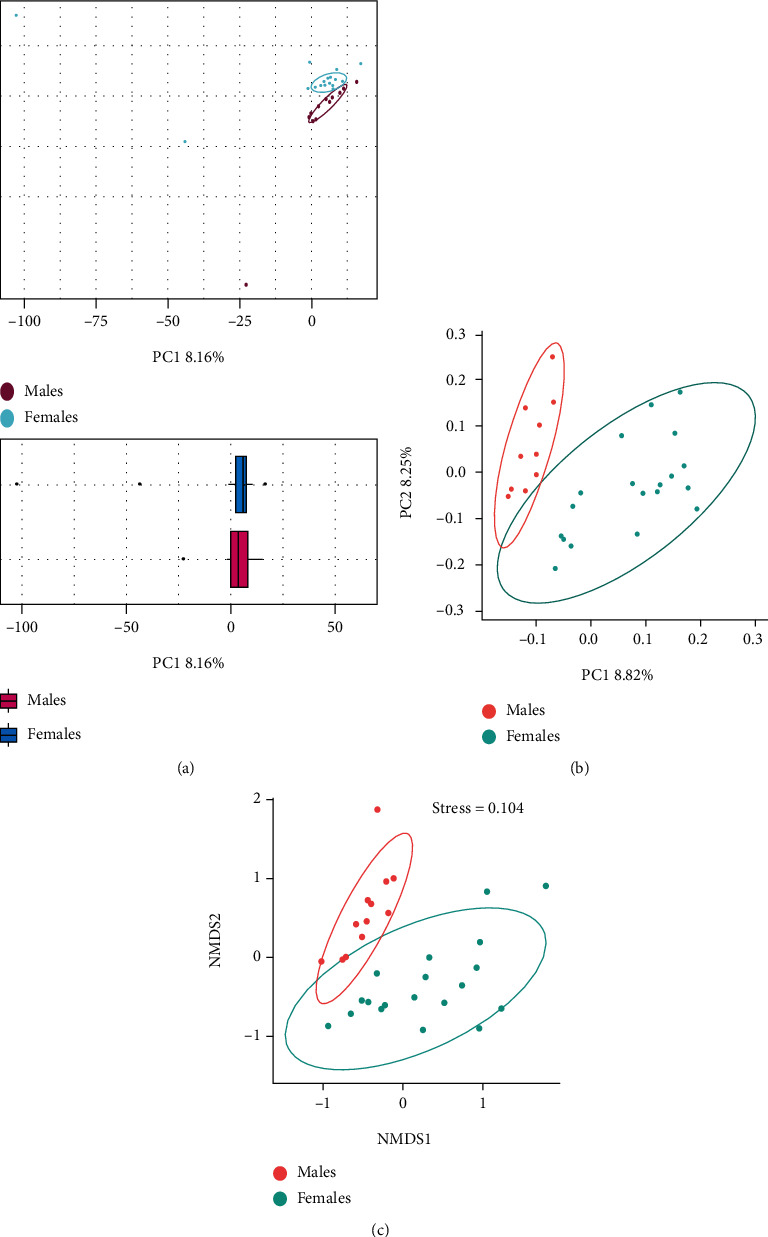
Similarity of microbial communities between elderly males and females with stage 1 periodontitis based on the unweighted UniFrac distance. (a) 2D diagram of principal component analysis (PCA). (b) 2D diagram of principal coordinate analysis. (c) 2D diagram of nonmetric multidimensional scale (NMDS) used to analyze the *β*-diversity of microbial communities between males and females.

Beta diversity refers to visualization of the differences in the diversity of microbiota in the two groups. The PCA (Figure 2(a)) and PCoA (Figure 2(b)) indicated the extent of the difference in the spatial distance, especially in PCoA analysis based on unweighted UniFrac distance. In NMDS analysis based on the binary Jaccard distance, we found a significant spatial distance and value of stress < 0.2, representing certain explanatory significance in the two-dimensional point graph (Figure 2(c)). The male and female groups were far apart (Figure 2), indicating that the oral microbiotas in elderly with initial periodontitis vary by gender.

### 3.3. Composition of the Oral Microbiota

**Figure 3:**
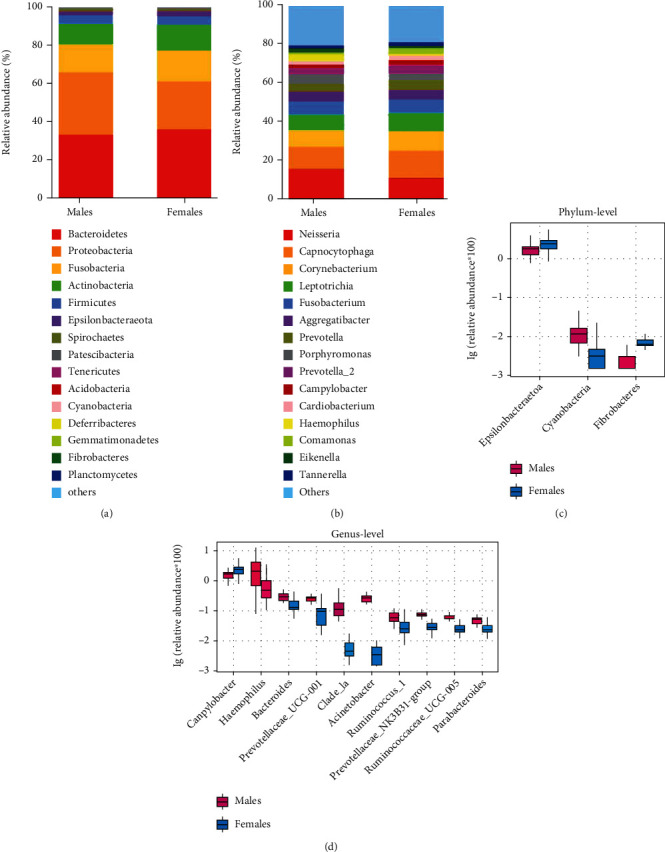
The oral microbiome composition of elderly males and females with stage 1 periodontitis. (a) Phylum level and (b) genus level composition. (c) Species difference analysis between males and females (group 2) by the Kruskal-Wallis test at the phylum and (d) genus levels.

**Figure 4:**
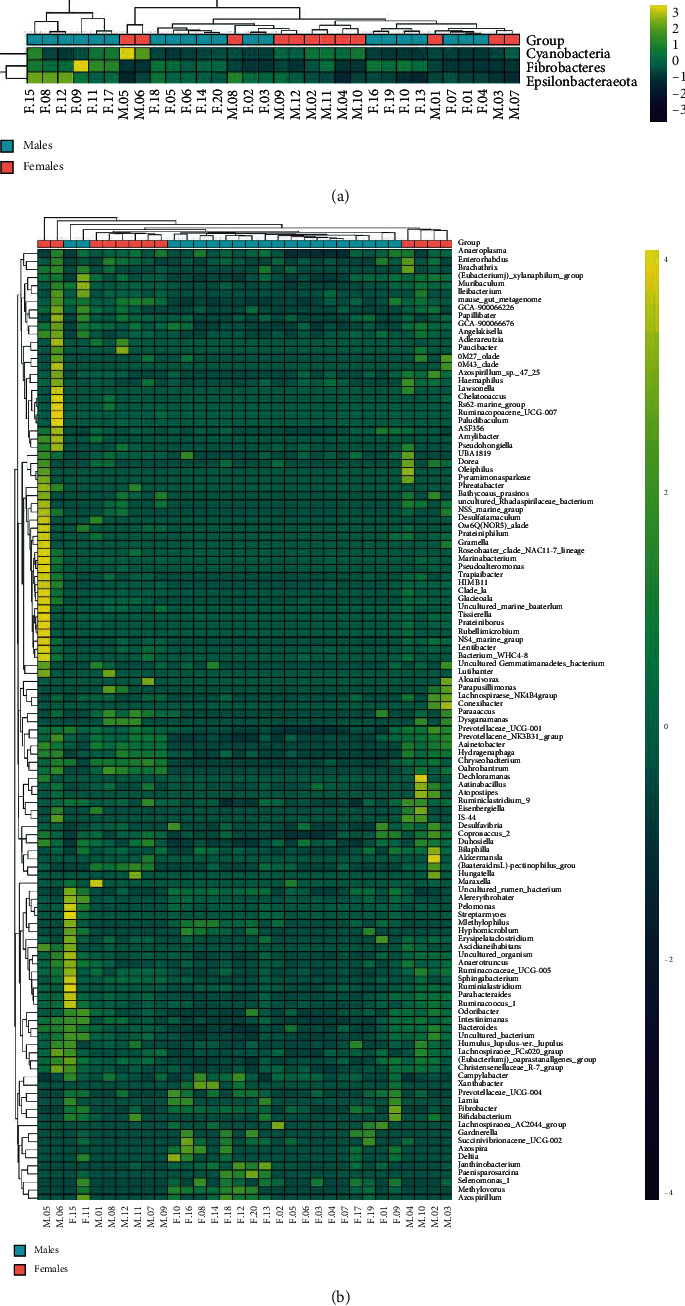
Heat map of differential oral microbiomes between elderly males and females with stage 1 periodontitis. (a) Phylum level. (b) Genus level. The sample information (group and number) and species annotation information are displayed on the horizontal axis and vertical axes, respectively. Colors indicate the Spearman rank correlation.

The relative abundance of the top 15 bacteria was assessed at the phylum level (Figure 3(a)) and genus level (Figure 3(b)). Multivariate analysis estimated further differences between the two groups and identified bacteria that differed significantly between them. A boxplot shows the top 3 different species at the phylum (Figure 3(c)) and top 10 different species at the genus (Figure 3(d)) levels. Compared to females, the relative abundance of cyanobacteria at the phylum level increased in males, whereas the relative abundance of Epsilonbacteraeota and Fibrobacteres was decreased (*P* < 0.05; Figure 3(c)). At the genus level, females had higher relative abundance of *Campylobacter* than males (*P* < 0.05). However, the relative abundance of *Haemophilus*, *Bacteroides*, *Prevotellaceae_UCG-001*, *Clade_Ia*, *Acinetobacter*, *Ruminococcus_1*, *Prevotellaceae_NK3B31_group*, *Ruminococcaceae_UCG-005*, and *Parabacteroides* was lower in females than in males (*P* < 0.05; Figure 3(d)).  Figure 4 shows the different species between the two groups in the form of a heat map.

**Figure 5:**
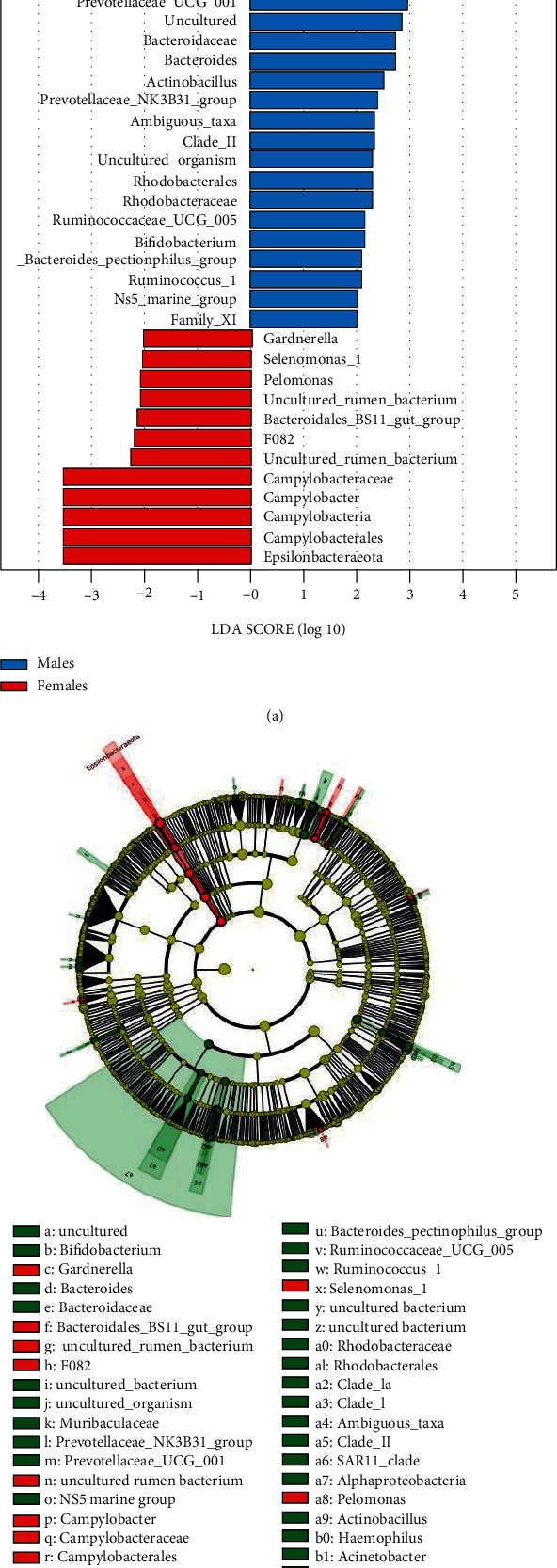
Different rich taxa between elderly males and females with stage 1 periodontitis analyzed by the linear discriminant analysis effect size. (a) Histogram of the LDA scores. (b) The phylogenetic tree in the form of a cladogram.

We conducted LEfSe to identify the differential bacterial composition between the male and female patients and screen for potential biomarkers. As stated above, significant differences in the microbiota were observed between males and females with initial periodontitis (Figure 5(a)). A cladogram showing the most discriminative bacterial clades identified by LEfSe is shown in Figure 5(b). The following species were more predominant in the supragingival plaques of men than women: the class Alphaproteobacteria, its order SAR11_clade or Rhodobacterales, its family Clade_I/Clade_II or Rhodobacteraceae, and its genus Clade_Ia/Ambiguous_tax, along with the family Muribaculaceae/Bacteroidaceae and the genus Prevotellaceae_UCG_001/Prevotellaceae_NK3B31_group. In males, genus *Haemophilus* had the largest LDA score. However, these results show the significantly higher level of the phylum Epsilonbacteraeota and the corresponding class Campylobacteria, order Campylobacterales, family Campylobacteraceae, and genus *Campylobacter*, in the subgingival microbiotas of females. The microbiotas of women were also enriched with genera mainly belonging to the Bacteroidetes phylum, particularly the F082 and Bacteroidales_BS11_gut_group family. In addition, the phylum Actinobacteria and its genus *Gardnerella*, along with the phylum Firmicutes and its genus Selenomonas_1, were high in females. The phylum Proteobacteria and its genus *Pelomonas* were also high in females. Detailed data and statistics are provided in Table 2.

### 3.4. Function Prediction

**Figure 6:**
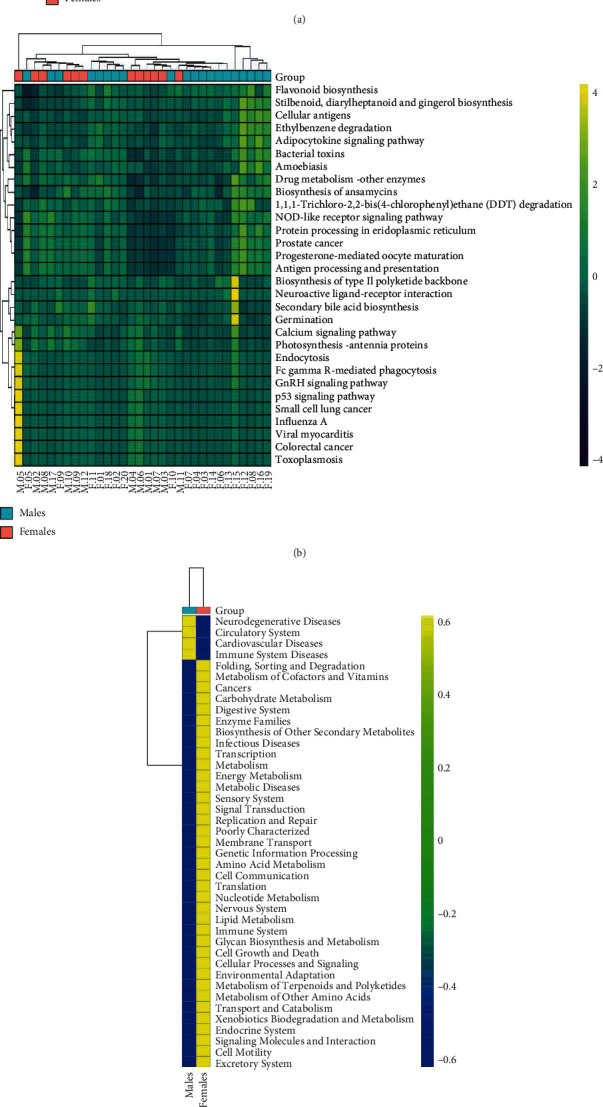
KEGG pathway enrichment analysis. (a) The different pathways in individual patients are shown in clustering heat maps at level 2 and (b) level 3 of KEGG. (c) At level 2 of KEGG, the different pathways between the two groups were clustered into a heat map.

By linking the genomes to pathways via KEGG orthologue annotation, we determined differences in the estimated bacterial functional capabilities in the 12 males and 20 females and compared them using QIIME2 and PICRUSt2. At the phylum level, the relative proportions of functions associated with immune system diseases, neurodegenerative diseases, the circulatory system, and cardiovascular diseases appeared to increase in male patients. However, the functions associated with immune response, including the immune system, increased in females, whereas the immune-related functions decreased in males (Figure 6).

## 4. Discussion

In this study, we performed a detailed analysis of deep sequencing data and showed that the diversity and abundance of oral bacteria varies significantly between elderly male and female patients with initial periodontitis. The *α*-diversity, reflecting species richness, was higher in males than females. The results of the *β*-diversity analysis showed that the samples were reasonably divided into different groups by gender. According to the relative abundance of species composition, LEfSe analysis showed that biomarkers in males were *Haemophilus*, family Muribaculaceae, and Clade_I/Clade_II, among others, whereas the dominant bacteria in females were *Campylobacter*, family F082, Bacteroidales_BS11_gut_group, *Selenomonas_1*, and *Pelomonas*. KEGG analysis showed that predicting changes in the female oral microbiota may be related to the immune system and immune system diseases are the main predictor of periodontitis in males.

Using the Kruskal-Wallis test for different species, the relative abundance of phyla Epsilonbacteraeota and Fibrobacteres and genus *Campylobacter* was higher in females than in males. The LEfSe analysis also identified *Campylobacter* as important. *Campylobacter* is Gram-negative microaerophilic bacteria that live as commensal organisms in the gastrointestinal tract. In addition to their own bacterial components, *Campylobacter* produces several different cytotoxins, including cytolethal distending toxin (CDT) and 1,3 galactosyltransferases involved in lipopolysaccharide (LPS) production, which play a role in colonization [29]. Lundmark et al. found that *Campylobacter conisus* is more abundant in healthy individuals through 16S rRNA sequencing of saliva samples from patients with chronic periodontitis and healthy periodontal controls [30]. An observational cross-sectional study of 76 postmenopausal women found *Campylobacter rectus* in the oral microflora of these subjects by real-time PCR [31]. Our results also showed that Campylobacter was abundant in the postmenopausal women compared with elderly men. Thus, the increase in *Campylobacter* may play a potential positive role in periodontal health in postmenopausal women. Interestingly, a previous study investigated the composition of the tongue microflora in 16 intraoral halitosis (IOH) patients and 10 healthy subjects and found that *Campylobacter* was significantly abundant in the IOH group [32]. This difference may be due to age, hormone levels, noncomparable study populations, and the diverse mechanisms of oral diseases.

An important factor affecting the oral microbiome is age [33]. The outer membrane vesicles or gingipains of *P. gingivalis* and free soluble bacterial components of *A. actinomycetemcomitans* released into the circulation can induce a proatherogenic responses in endothelial cells [34], suggesting that the microbiota plays a role in cardiovascular disease in older adults. This may be associated with a general decrease in immune function and the development of chronic inflammation during aging. In men, age correlates with a more pronounced decrease in the total number of T and B cells and larger increase in senescent CD8+T effector memory cells compared to women [35, 36]. In contrast, menopause is associated with increased levels of IL-1*β*, IL-6, and TNF-*α* (proinflammatory) and reduced levels of IFN-*γ* (anti-inflammatory). In aged women, monocytes have a proinflammatory phenotype and NK cell robust cytotoxic activity [37]. Furthermore, periodontitis has been reported to occur more often in postmenopausal women who do not receive hormone replacement compared to premenopausal women [31]. Our results showed that *Haemophilus* had the largest LDA score in males. *Haemophilus* is a Gram-negative bacteria that can only grow with fresh blood during artificial culture. It is deposited mainly in the throat and oral mucosa of humans and animals, which could cause primary suppurative infection and serious secondary infection [38]. The main virulence factors include capsule and lipooligosaccharide (LOS). The LOS of *Haemophilus ducreyi* induces immunosuppressive enzyme expression in DCs, largely through type I IFN- and TNF-*α*-dependent mechanisms, as well as the MAPK, NF-*κ*B, and JAK-STAT pathways [39]. Other studies have shown that *Haemophilus* is related to sex differences. A 16S rRNA gene sequencing analysis of sputum samples from patients with asthma and normal controls found that *Haemophilus* spp. are associated with asthma in men but not in women [40]. On the other hand, in contrast to the present study, Raju et al. found a high relative abundance of *Haemophilus* in the saliva microbiota of females [41]. The LEfSe analysis identified additional biomarkers, including Rhodobacteraceae, Actinobacillus, Prevotellaceae, Muribaculaceae, and Bacteroidaceae. The immunomodulatory effects of LPS from *Rhodopseudomonas sphaeroides* are mainly the result of eliminating the inhibitory effects of T cells; this permitted the positive effects of amplifier T cells to be more fully expressed, resulting in an increased antibody response [42]. Furthermore, *Actinobacillus actinomycetemcomitans*, one of the major causative agents of chronic inflammatory periodontal disease, has been shown to cause a specific immune response by the host [43].

The dominant bacteria mentioned above may change the host immune response. We used KEGG to predict signaling pathways involved by the bacteria. Immune system diseases, neurodegenerative diseases, circulatory system diseases, and cardiovascular diseases were enriched in males, whereas immune system diseases, infectious diseases, metabolic diseases, and endocrine system diseases were increased in females. Jansen et al. have indicated that female-biased Gene Ontology categories are highly enriched for various immune system functions, including the TLR3 and TLR4 pathways, as well as genes linked to autoimmune diseases and genes regulated by estrogen and LPS [44]. As mentioned above, *Campylobacter* is a commensal organism in the gastrointestinal tract. Commensal microorganisms are greatly involved in maintaining homeostasis and health not only by blocking microbial activity but also by reinforcing the human immune system via specialized mechanisms [45], which further indicates a correlation between the oral microbiome and immune system. In KEGG pathway analysis, the endocrine system was enriched in females. The differences may be related to the change in female sexual hormones throughout life, one of the factors that plays an essential role in microbiota modulation. With increasing age, estrogen and androgen levels vary in both males and females with some commonality, as well as significant differences [46]. Though estrogen levels drastically decrease with menopause in women, androgens progressively decrease in both sexes starting at approximately 30 years of age [47–49]. Changes in periodontal status have also been found to be associated with variations in levels of sex hormones [50]. Finally, specific bacterial species, such as *P. gingivalis* and *Tannerella forsythensis*, have been shown to be important in postmenopausal women regarding the etiology of periodontitis [51].

## 5. Conclusion

Overall, our findings indicate that gender may be a differentiating factor in the microbial composition of the subgingival plaques of elderly patients with initial periodontitis. Future oral microbiome studies may yield better resolution if the context of sex-specific differences is considered.

## Figures and Tables

**Table 1:** Age information of the enrolled participants.

Group	Number	Age (year)
Group 1	12	54.00 ± 3.54
Group 2	20	58.55 ± 7.45
*P*		>0.05

**Table 2:** Linear discriminant analysis (LDA) effect size (LEfSe) results of the distinct taxa between males and females.

Biomarker	Groups	LDA value
Bacteria.Proteobacteria.Gammaproteobacteria.Pasteurellales.Pasteurellaceae.Haemophilus	Males	4.05
Bacteria.Bacteroidetes.Bacteroidia.Bacteroidales.Muribaculaceae	Males	3.46
Bacteria.Bacteroidetes.Bacteroidia.Bacteroidales.Muribaculaceae.uncultured_bacterium	Males	3.40
Bacteria.Proteobacteria.Alphaproteobacteria	Males	3.28
Bacteria.Proteobacteria.Gammaproteobacteria.Pseudomonadales	Males	3.33
Bacteria.Proteobacteria.Alphaproteobacteria.SAR11_clade	Males	3.25
Bacteria.Proteobacteria.Alphaproteobacteria.SAR11_clade.Clade_I	Males	3.18
Bacteria.Proteobacteria.Alphaproteobacteria.SAR11_clade.Clade_I.Clade_Ia	Males	3.16
Bacteria.Bacteroidetes.Bacteroidia.Bacteroidales.Bacteroidaceae.Bacteroides	Males	2.72
Bacteria.Bacteroidetes.Bacteroidia.Bacteroidales.Bacteroidaceae	Males	2.72
Bacteria.Proteobacteria.Gammaproteobacteria.Pseudomonadales.Moraxellaceae	Males	3.12
Bacteria.Bacteroidetes.Bacteroidia.Bacteroidales.Prevotellaceae.Prevotellaceae_UCG_001	Males	2.92
Bacteria.Proteobacteria.Gammaproteobacteria.Pseudomonadales.Moraxellaceae.Acinetobacter	Males	3.10
Bacteria.Patescibacteria.Gracilibacteria.Absconditabacteriales__SR1_.uncultured_bacterium	Males	3.02
Bacteria.Patescibacteria.Gracilibacteria.Absconditabacteriales__SR1_.uncultured_bacterium.uncultured_bacterium	Males	3.02
Bacteria.Actinobacteria.Actinobacteria.Actinomycetales.Actinomycetaceae.Uncultured	Males	2.84
Bacteria.Bacteroidetes.Bacteroidia.Bacteroidales.Muribaculaceae.uncultured_organism	Males	2.27
Bacteria.Bacteroidetes.Bacteroidia.Bacteroidales.Prevotellaceae.Prevotellaceae_NK3B31_group	Males	2.39
Bacteria.Proteobacteria.Gammaproteobacteria.Pasteurellales.Pasteurellaceae.Actinobacillus	Males	2.51
Bacteria.Firmicutes.Clostridia.Clostridiales.Ruminococcaceae.Ruminococcus_1	Males	2.07
Bacteria.Firmicutes.Clostridia.Clostridiales.Ruminococcaceae.Ruminococcaceae_UCG_005	Males	2.14
Bacteria.Proteobacteria.Alphaproteobacteria.Rhodobacterales.Rhodobacteraceae	Males	2.27
Bacteria.Proteobacteria.Alphaproteobacteria.Rhodobacterales	Males	2.27
Bacteria.Proteobacteria.Alphaproteobacteria.SAR11_clade.Clade_II	Males	2.31
Bacteria.Proteobacteria.Alphaproteobacteria.SAR11_clade.Clade_II.Ambiguous_taxa	Males	2.32
Bacteria.Actinobacteria.Actinobacteria.Bifidobacteriales.Bifidobacteriaceae.Bifidobacterium	Males	2.13
Bacteria.Firmicutes.Clostridia.Clostridiales.Family_XI	Males	2.01
Bacteria.Firmicutes.Clostridia.Clostridiales.Lachnospiraceae._Bacteroides__pectinophilus_group	Males	2.10
Bacteria.Bacteroidetes.Bacteroidia.Flavobacteriales.Flavobacteriaceae.NS5_marine_group	Males	2.03
Bacteria.Epsilonbacteraeota	Females	3.55
Bacteria.Epsilonbacteraeota.Campylobacteria.Campylobacterales	Females	3.55
Bacteria.Epsilonbacteraeota.Campylobacteria	Females	3.55
Bacteria.Epsilonbacteraeota.Campylobacteria.Campylobacterales.Campylobacteraceae.Campylobacter	Females	3.53
Bacteria.Epsilonbacteraeota.Campylobacteria.Campylobacterales.Campylobacteraceae	Females	3.53
Bacteria.Bacteroidetes.Bacteroidia.Bacteroidales.F082	Females	2.18
Bacteria.Bacteroidetes.Bacteroidia.Bacteroidales.F082.uncultured_rumen_bacterium	Females	2.08
Bacteria.Proteobacteria.Gammaproteobacteria.Betaproteobacteriales.Burkholderiaceae.Pelomonas	Females	2.06
Bacteria.Actinobacteria.Actinobacteria.Bifidobacteriales.Bifidobacteriaceae.Gardnerella	Females	2.00
Bacteria.Bacteroidetes.Bacteroidia.Bacteroidales.Bacteroidales_BS11_gut_group	Females	2.13
Bacteria.Firmicutes.Negativicutes.Selenomonadales.Veillonellaceae.Selenomonas_1	Females	2.00
Bacteria.Bacteroidetes.Bacteroidia.Bacteroidales.uncultured.uncultured_rumen_bacterium	Females	2.25

Only an LDA score of >2.0 is shown.

## Data Availability

Raw reads have been deposited at NCBI under the BioProject accession number PRJNA763744.
